# miR-26a enhances autophagy to protect against ethanol-induced acute liver injury

**DOI:** 10.1007/s00109-015-1282-2

**Published:** 2015-04-17

**Authors:** Weidong Han, Xianghui Fu, Jiansheng Xie, Zhipeng Meng, Ying Gu, Xichun Wang, Ling Li, Hongming Pan, Wendong Huang

**Affiliations:** Department of Medical Oncology, Sir Run Run Shaw Hospital, College of Medicine, Zhejiang University, 3 East Qingchun Road, Hangzhou, Zhejiang 310016 China; Division of Molecular Diabetes Research, Department of Diabetes and Metabolic Diseases Research, Beckman Research Institute, City of Hope National Medical Center, 1500 E. Duarte Road, Duarte, CA 91010 USA; Division of Endocrinology and Metabolism, State Key Laboratory of Biotherapy, West China Hospital, Sichuan University, Chengdu, 610041 Sichuan China; Collaborative Innovation Center of Biotherapy, Chengdu, 610041 Sichuan China

**Keywords:** miR-26a, Autophagy, Hepatic steatosis, Mitogen-activated protein kinases, Ethanol binge

## Abstract

**Abstract:**

Autophagy is a process for the turnover of intracellular organelles and molecules during stress responses. microRNAs (miRNAs) are small, non-coding endogenous RNAs that may regulate almost every cellular process. However, the roles of miRNAs in autophagy are still poorly understood. In this study, we show that miR-26a enhances autophagy in both culture cells and the mouse liver. Hepatic overexpression of miR-26a in mice alleviated ethanol-induced hepatic steatosis and liver injury. Overexpression of miR-26a increased the expression of the autophagy mediator Beclin-1, which is regulated by mitogen-activated protein kinases (MAPKs). We identified DUSP4 and DUSP5, two MAPKs inhibitors, as direct targets of miR-26a. We further demonstrated that miR-26a targeted the 3′-UTRs of several other negative regulators of autophagy. Our results thus identify a novel miRNA-mediated mechanism that enhances cytoprotective autophagy in the liver.

**Key messages:**

• miR-26a enhances autophagy in liver cells.

• Hepatic overexpression of miR-26a in mice alleviates ethanol-induced liver injury.

• Overexpression of miR-26a increases the expression of autophagy mediator Beclin-1.

• DUSP4 and DUSP5, two MAPKs inhibitors, were identified as direct targets of miR-26a.

**Electronic supplementary material:**

The online version of this article (doi:10.1007/s00109-015-1282-2) contains supplementary material, which is available to authorized users.

## Introduction

Macroautophagy (hereafter referred to as autophagy) is a bulk intracellular degradation system that is responsible for the turnover of long-lived proteins, cytosolic components, and damaged organelles [1]. Autophagy occurs at low levels in all cells to maintain cellular homeostasis, via processes that include the turnover of misfolded proteins and damaged organelles. However, under cellular stress conditions, such as a nutrient-deficient environment, autophagy is rapidly activated to provide an alternative source of energy to enable cells to survive. The molecular mechanism of autophagy involves several conserved autophagy-related proteins (ATGs). Beclin-1, the mammalian orthologue of yeast ATG6, has a central role in autophagy. It interacts with several cofactors to regulate the lipid kinase Vps-34 protein and promote the formation of Beclin-1–Vps34–Vps15 core complexes, thereby inducing autophagy [2]. Therefore, regulation of Beclin-1 is an important mechanism to control autophagy under physiological and pathological conditions. It has been reported that activation of the mitogen-activated protein kinase (MAPK) family, including ERK, p38, and JNK, leads to an increase in Beclin-1 expression, which in turn results in cytoprotective autophagy [3].

Autophagy is also known to be widely involved in the pathogenesis of diseases and is activated under a variety of stress conditions. For example, it is activated in response to ethanol exposure. This ethanol-induced autophagy in the liver is important to avert the pathologic effects of ethanol metabolism. Pretreatment of mice with rapamycin, a well-known autophagy inducer, dramatically reduced the number of lipid droplets (LDs) in the ethanol-treated livers. These results indicate that autophagy plays an important role in alleviating ethanol-induced hepatic steatosis and liver injury [4]. The underlying mechanism for ethanol-induced autophagy remains poorly understood, although a recent study showed that FOXO3 may play a role in this process [5].

microRNAs (miRNAs) are 20- to 22-nucleotide non-coding RNAs that repress the expression of their cognate target genes by specifically binding and cleaving messenger RNAs (mRNAs), inhibiting translation, and/or deadenylating mRNA tails [6]. miRNAs have been shown to control various fundamental biological processes, including cell proliferation, apoptosis, and autophagy [7–9]. The regulatory roles of miRNAs in autophagy were first uncovered in 2009 when Beclin-1, an important autophagy-promoting gene, was shown to be post-transcriptionally modulated by miR-30a [10]. Soon after this report, a number of miRNAs were characterized to modulate some members of ATGs and their regulators at different autophagic stages. Importantly, these miRNAs have been associated with certain diseases, including cancers, cardiac pathologies, bacterial infection, and Crohn’s disease [11–14].

miR-26a is completely conserved across vertebrates. It plays a dual role in different cancers, either as a tumor suppressor [15–17] or a tumor promoter [18, 19]. miR-26a also plays important roles in regulating IFN-β anti-inflammatory signaling [20], HASMC hypertrophy [21], pancreatic cell differentiation [22], pathological and physiological angiogenesis [23], hepatocyte proliferation during liver regeneration [24], and other processes. However, the role of miR-26a in autophagy remains unknown. Recent studies have demonstrated that hypoxia activates autophagy [25]. Interestingly, miR-26a is induced by hypoxia and can decrease proapoptotic signaling in a hypoxic environment [26], suggesting a potential role of miR-26a in autophagy.

In the present study, we investigated the potential role of miR-26a in modulating autophagy and acute alcoholic liver injury in mice. Our study demonstrated that miR-26a expression was significantly increased during the autophagic process. The overexpression of miR-26a promoted autophagy in both culture cells and the mouse liver. More importantly, liver-specific overexpression of miR-26a protected the mice against ethanol-induced acute liver injury through the upregulation of autophagy. Our findings thus identify a novel miRNA-mediated mechanism for enhancing cytoprotective autophagy and provide a new approach to treat ethanol-induced liver injury.

## Materials and methods

### Mice

To generate liver-specific miR-26a transgenic (L-TG) mice, a genomic DNA fragment encoding the miR-26a-1 locus, preceded by the synthetic CAG promoter and a loxP-flanked Neo-STOP cassette, was inserted into the Rosa26 locus. Mice were generated by injecting targeted ES cells into blastocysts and maintained in a mixed C57BL/6 and 129 background [22]. Mice carrying the targeted allele were bred with Alb-Cre mice, which selectively deleted the “Neo-STOP” cassette in hepatocytes. Heterozygous miR-26a transgenic mice and littermate wild-type mice were used for experiments. All procedures followed the National Institutes of Health guidelines for the care and use of laboratory animals.

The ethanol binge was conducted as previously described [4]. Briefly, after 6 h of fasting, mice were given 33 % (vol/vol) ethanol at a total accumulative dosage of 4.5 g/kg body weight by three equally divided gavages at 20-min intervals. Control mice received the same volume of water. For autophagy inhibition, chloroquine (CQ; 60 mg/kg) was given (intraperitoneally) to the mice 30 min before the administration of ethanol. Mice were analyzed 16 h later.

### In vivo delivery of locked nucleic acid -modified anti-miR-26a

The locked nucleic acid (LNA)–anti-miR26a oligonucleotides were purchased from Exiqon (Denmark). Five 6-week-old 129 mice per group were injected intraperitoneally with 10 mg/kg of anti-miR-26a or vehicle control every 2 days for a total of four injections.

### miR-26a and LNA miR-26a inhibitor transfections

The miRNA mimic and miRCURY LNA miR-26a inhibitor were purchased from Ambion (Austin, TX, USA) and Exiqon (Vedbaek, Denmark), respectively. Transfections of miRNA or inhibitors were performed using HiPerFect (Qiagen, Valencia, CA, USA) according to the manufacturer’s protocol.

### Renilla luciferase-based screening assay

SK-Hep-1 cells with stable expression of renilla luciferase (RLuc)–LC3^WT^ or RLuc–LC3^G120A^ were reverse-transfected side by side in 96-well format with 20 nM of miRNA or siRNA. Transfections were performed using HiPerFect (Qiagen). At 22 h after transfection, 50 nM of EnduRen substrate (Promega, Madison, WI, USA) was added. RLuc activity was measured at 24 and 48 h after transfection. Luciferase measurements were performed using the DTX 800 Multimode Detector (Beckman Coulter, Fullerton, CA).

### Luciferase activity assays

Hela cells were transfected with 40 nM miRNA precursors (GenePharma, Shanghai, China) and 200 ng of psicheck2.2 (Promega) constructs containing an insert of the 3′ untranslated region (3′-UTR) or flanking sequences of seed nucleotides of miR-26a target genes using Attractene (Qiagen) in 96-well plates. At 24 h after transfection, cells were analyzed with a Dual-Luciferase Reporter Assay (Promega). For mutant reporter constructs, the seed sequence in the 3′-UTR, 5′-TACTTGA-3′, was mutated to 5′-ATGATGA-3′.

### Serum alanine aminotransferase and hepatic triglyceride analysis

Serum was obtained by centrifuging whole mouse blood (1300*g* for 10 min) at 4 °C. Levels of serum alanine aminotransferase (ALT) were measured at the City of Hope Helford Research Hospital. To quantify the hepatic triglyceride content, liver tissues (100 mg) were homogenized in ice-cold buffer containing 20 mmol/L Tris-HCl, 150 mmol/L NaCl, 2 mmol/L EDTA, and 1 % Triton X-100, pH 7.5. The triglyceride content of this 100-μL solution was determined at the City of Hope Helford Research Hospital.

### Statistical analysis

Data are expressed as mean ± SD. A two-tailed Student’s *t* test was used to determine the differences between two data groups. *P* < 0.05 was considered as statistically significant.

### Other methods

Please see supplementary “Materials and methods”.

## Results

### miR-26a is regulated during the autophagic process

Nutrient deprivation and rapamycin treatment are known to activate autophagy in various types of cells. To explore the potential involvement of miR-26a in autophagy, we measured the endogenous miR-26a expression upon autophagy induction in several cell lines, including SK-Hep-1, Huh 7, HepG2, and Hela. Quantitative RT-PCR (qRT-PCR) analysis revealed that miR-26a was significantly upregulated in cells treated with autophagic stimuli compared to the untreated controls (supplementary Fig. 1a), which suggests the induction of miR-26a by the autophagic process. We further examined miR-26a expression in SK-Hep-1 cells over different time courses of autophagy. Supplementary Fig. 1b shows that miR-26a expression was gradually increased by HBSS or rapamycin treatment. These results suggest a potential role for this miRNA in response to autophagic stimuli.

### Overexpression of miR-26a induces autophagic activity

**Fig. 1:**
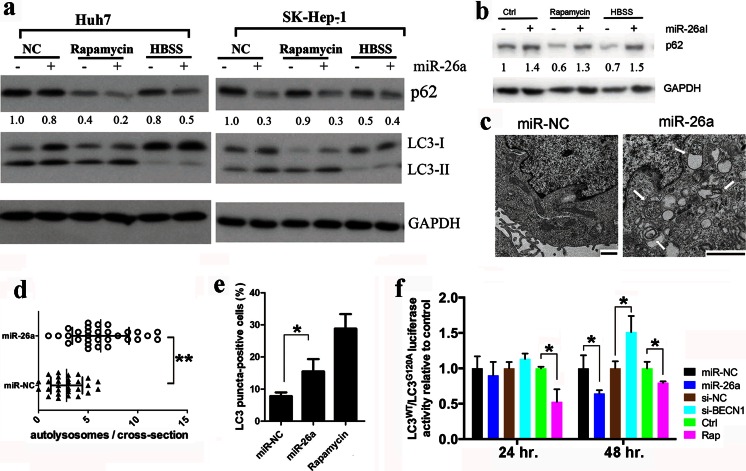
miR-26a overexpression promotes autophagy. **a** Cells were transfected with miR-26a or scramble miRNA (miR-NC) for 24 h. Then, cells were treated with HBSS for 4 h or 125 nM rapamycin for 24 h. The expression of p62 and LC3 was detected by immunoblotting. **b** SK-Hep-1 cells were transfected with miR-26a inhibitor (miR-26aI) or negative control for 24 h. Then, cells were treated with HBSS for 4 h or 125 nM rapamycin for 24 h. The expression of p62 was detected by immunoblotting. The relative quantity of p62 in **a** and **b** was calculated by ImageJ densitometric analysis and normalized using GAPDH. **c** Representative TEM images of SK-Hep-1 cells transfected with miR-26a for 48 h as indicated. *Bar* 1 μm. *Arrows* denote autolysosomes. **d** Quantification of autolysosomes (denoted by *white arrows*) per cell (*n* = 30). **e** Percentage of red or yellow puncta-positive cells was quantified by automated image acquisition and analysis using a threshold of more than five dots per cell. **f** SK-Hep-1 RLuc–LC3^WT^ and RLuc–LC3^G120A^ cells were reverse-transfected with miRNAs and siRNAs or treated with 125 nM rapamycin. Luciferase activity was measured at 24 and 48 h after transfection or rapamycin treatment. Results shown are the mean ± SD of at least three independent experiments. *P < 0.05; ** *P* < 0.01

Starvation- and rapamycin-induced miR-26a expression could be a cause or merely a consequence of autophagy. To clarify this possibility, we employed four independent approaches to investigate the effects of miR-26a on autophagy. First, the levels of LC3 and p62/SQSTM1, two classic markers of autophagy, were determined by immunoblot analysis. As shown in Fig. 1a, LC3-II, a PE-conjugated form of LC3, which is converted from LC3-I upon autophagy induction, was significantly increased in cells transfected with miR-26a precursors. By contrast, p62, a selective substrate of autophagy and a biomarker for autophagic flux, was significantly decreased in cells transfected with miR-26a under normal or autophagic conditions. These results indicate that overexpression of miR-26a activates autophagy in cells. In addition, inhibition of endogenous miR-26a expression by transfection of anti-miR-26a increased the expression of p62 in SK-Hep-1 cells under normal or stressful conditions (Fig. 1b).

In the second approach, ultrastructural analysis by transmission electron microscopy (TEM) showed that overexpression of miR-26a in SK-Hep-1 cells resulted in increased autophagic vacuoles containing organelle remnants (Fig. 1c). Quantification of autolysosomes per cellular cross-section revealed a significant increase upon miR-26a overexpression, relative to the scramble control (Fig. 1d), thereby confirming our findings described above.

Third, to separately evaluate the extent of autophagosome and autolysosome accumulation, we used SK-Hep-1 cells that stably express the tandem fluorescent mRFP-GFP-LC3 plasmid (SK-Hep-1/tf-LC3) [27]. mRFP retains its fluorescence, even in the acidic environment of lysosomes, whereas GFP loses its fluorescence. Thus, green LC3 puncta primarily indicate autophagosomes, whereas red LC3 puncta indicate both autophagosomes and autolysosomes. The red puncta that overlay with the green puncta and appear yellow in merged images are indicators of autophagosomes, whereas the free red puncta that do not overlay with the green puncta and appear red in merged images are indicative of autolysosomes. The numbers of yellow and red puncta were both significantly increased after miR-26a transfection (supplementary Figs. 2 and Fig. 1e), indicating that miR-26a enhanced the formation of both autophagosomes and autolysosomes in SK-Hep-1 cells.

Finally, the effect of miR-26a on autophagic flux was further examined using a functional screening approach as previously reported [11, 28]. In this system, LC3 is fused to a renilla luciferase reporter forming the RLuc–LC3 fusion protein. As LC3 itself is specifically degraded by autophagy, the level of autophagy in the SK-Hep-1 reporter cell line stably expressing wild-type RLuc–LC3 (RLuc–LC3^WT^) can be measured in real time using the in vivo renilla luciferase substrate EnduRen^TM^ [28]. As controls, SK-Hep-1 cells expressing a mutant fusion protein, RLuc–LC3^G120A^, which is unable to undergo autophagosomal localization and is not degraded by autophagy, are assayed in parallel. Thus, the autophagic flux can be evaluated as a change in the relative levels of these two fusion proteins (LC3^WT^/LC3^G120A^). In this assay, an siRNA against Beclin-1, which is a key regulator of autophagy formation, was used as a control for autophagy inhibition, while rapamycin was used as a positive control for autophagy induction. As expected, the ratio of LC3^WT^/LC3^G120A^ was increased by the inhibition of Beclin-1 but decreased with rapamycin treatment. Interestingly, the ratio of LC3^WT^/LC3^G120A^ was decreased significantly when cells were transfected with miR-26a for 48 h (Fig. 1f), indicating that miR-26a accelerates autophagic flux and stimulates the degradation of LC3. Collectively, these results demonstrate that miR-26a enhances autophagy in liver cells.

### miR-26a promotes autophagy as a protection mechanism against ethanol-induced acute liver injury

**Fig. 2:**
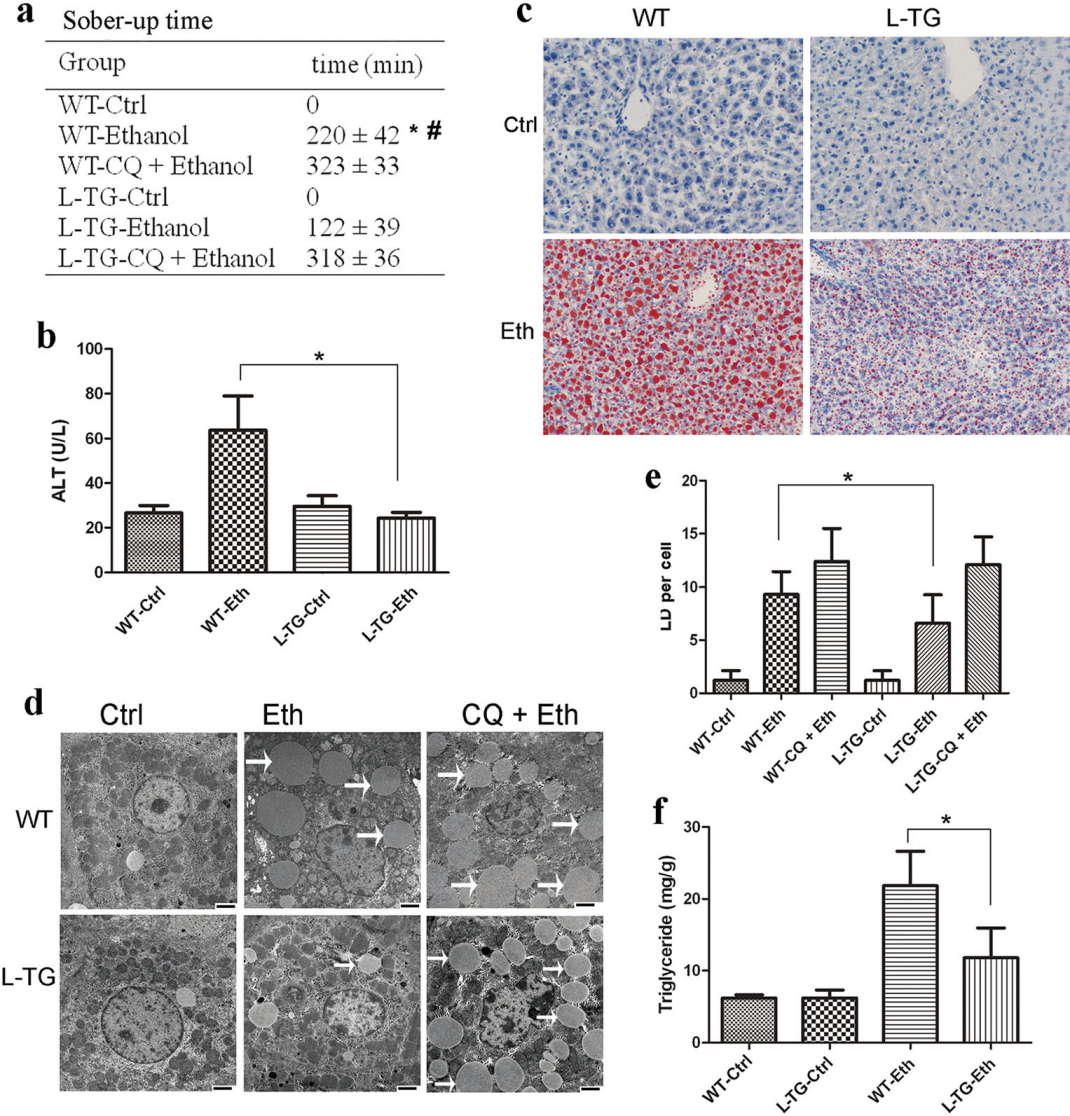
Overexpression of miR-26a alleviates ethanol-induced hepatic steatosis and liver injury in mice by the upregulation of autophagy. Wild-type (*WT*) or liver-specific miR-26a transgenic mice (*L-TG*) were treated with ethanol (*Eth*) or control vehicle (*Ctrl*). **a** Sober-up time of mice after ethanol binge. *P < 0.05 compared with the L-TG-ethanol group; #*P* < 0.05 compared with the WT-CQ + ethanol group. **b** Blood ALT level. **c** Cryosections of livers were stained with Oil Red O. **d**, **e** Liver samples were examined by TEM, and the number of lipid droplets (denoted by *white arrows*) per cell was quantified by counting the number of LDs per cross-sectioned cell. *Scale bar* 2 μm. **f** The total hepatic triglyceride levels of mice were determined; *n* = 4–5 mice per group. Results shown are the mean ± SD. *P < 0.05

Autophagy plays an important role in alleviating ethanol-induced hepatic steatosis and liver injury [4]. Therefore, we asked whether increased miR-26a expression could protect mice against acute alcohol-induced liver injury. To address this question, we utilized recently established miR-26a liver-specific overexpression mice (L-TG), in which the expression of miR-26a is elevated approximately 30 times compared with WT littermates (supplementary Fig. 3). Mice were gavaged intragastrically with ethanol, and the sober-up time (time between loss of righting reflex to recovery) was recorded. As shown in Fig. 2a, the sober-up time of L-TG mice is significantly less than that of WT mice, with 2 h in L-TG mice and 3.5 h in WT mice. Accordingly, ethanol-induced liver injury, as reflected by the serum ALT levels, was strongly reduced in L-TG mice (Fig. 2b). Autophagy likely protects hepatocytes against the detrimental effects of ethanol by removing damaged mitochondria and accumulated lipid droplets [4]. Oil Red O staining showed that ethanol-induced LDs levels were significantly lower in L-TG mice than that in WT mice (Fig. 2c). Compared to the WT livers, the quantification of LDs by TEM further confirmed a significant decrease in LDs in the L-TG livers (Fig. 2d, e). Consistently, ethanol-induced increases of hepatic triglyceride levels were significantly diminished in L-TG mice compared with WT mice (Fig. 2f). Hematoxylin and eosin (H&E) staining also indicated that WT mice exhibit higher grades of steatosis than L-TG mice when treated with ethanol (supplementary Fig. 4).

We then investigated whether the protective effect of L-TG mice is associated with autophagy. To this end, we pretreated L-TG and WT mice with the lysosome inhibitor CQ, a well-known late-phase autophagy inhibitor. CQ pretreatment significantly increased the sober-up time of both genotypes after ethanol binge (Fig. 2a). Strikingly, similar to WT mice, L-TG mice pretreated with CQ could not sober up until 5 h after the ethanol binge (Fig. 2a). Blockage of lysosomal function with CQ significantly increased the number of LDs in hepatocytes. Importantly, hepatocytes from both L-TG and WT mice pretreated with CQ exhibited no difference in LDs (Fig. 2d, e). H&E staining also revealed that ethanol-induced liver steatosis or injury in mice of both genotypes was further exacerbated by CQ combine treatment (supplementary Fig. 4). All of these findings suggest that the protective function of miR-26a on hepatocytes depends on autophagy.

**Fig. 3:**
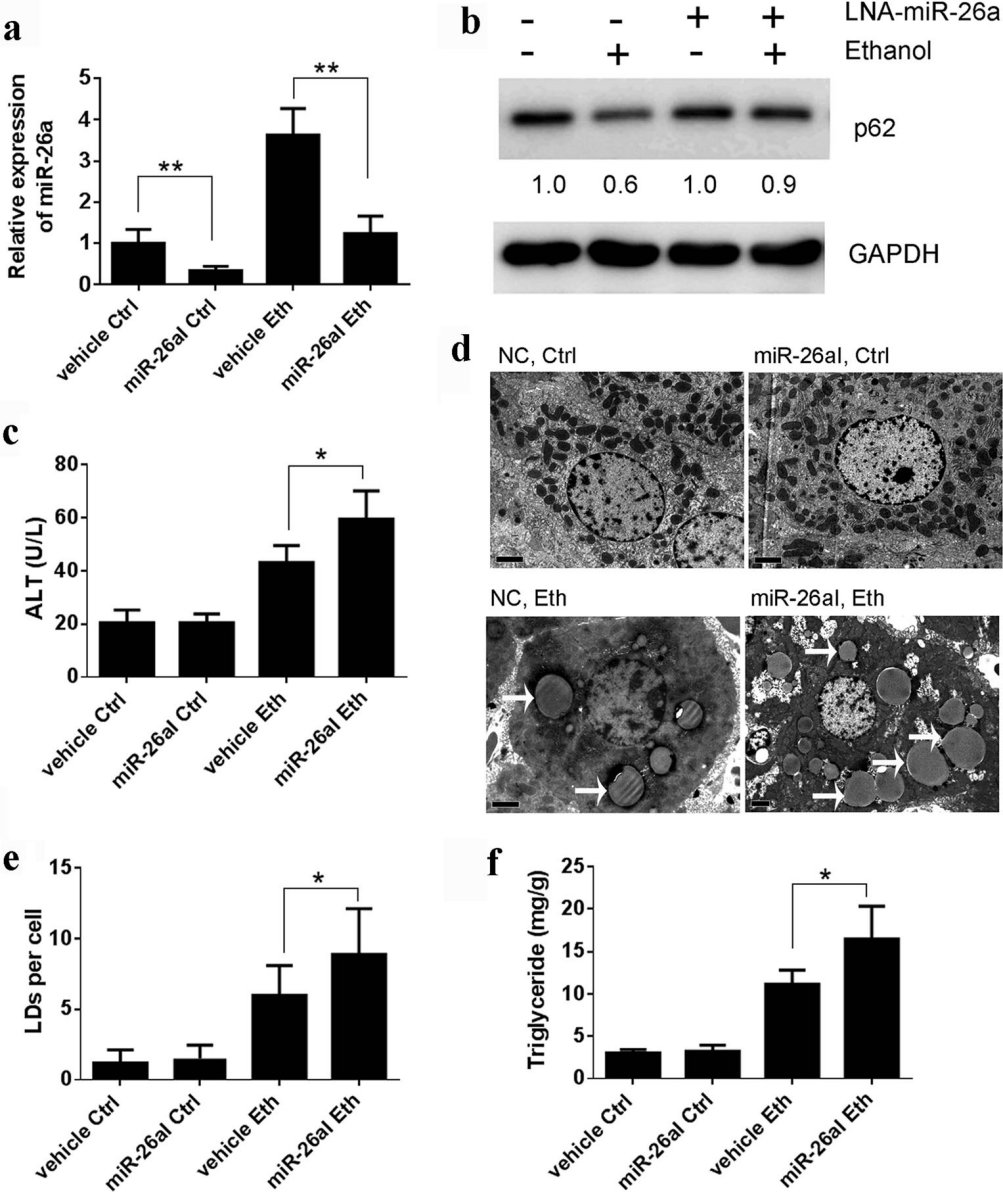
Inhibition of endogenous miR-26a-enhanced ethanol-induced liver injury. Mice were gavaged with ethanol (*Eth*) or water (*Ctrl*) in the presence or absence of anti-miR-26a (*miR-26aI*). **a** Expression of miR-26a in the mouse liver was analyzed by qRT-PCR. **b** Liver lysate was subjected to immunoblotting for p62. Each *lane* represents lysate from five mice. The relative quantity was calculated by ImageJ densitometric analysis and normalized using GAPDH. **c** Blood ALT level was analyzed. **d**, **e**. Liver samples were examined by TEM, and the number of LDs (denoted by *white arrows*) per cell was quantified by counting the number of LDs per cross-sectioned cell. *Scale bar* 2 μm. **f** The total hepatic triglyceride levels of mice were determined; *n* = 5 mice per group. Results shown are the mean ± SD. *P < 0.05; **P < 0.01

To further verify the protective role of miR-26a in ethanol-induced liver injury, we used locked nucleic acid-modified anti-miR-26a (LNA–anti-miR-26a) to inhibit endogenous miR-26a expression. As shown in Fig. 3a, the ethanol binge induced an approximately three- to fourfold increase of miR-26a expression in the liver, and the expression of miR-26a was successfully inhibited by LNA–anti-miR-26a. Pretreatment of mice with a miR-26a inhibitor reduced the ethanol binge-induced autophagy flux in the liver as shown by the increased levels of p62 (Fig. 3b) and significantly enhanced ethanol-induced liver injury as shown by the increased blood ALT levels (Fig. 3c). Furthermore, the miR-26a inhibitor-treated mice exhibited a significantly increased number of LDs (Fig. 3d, e) and the levels of hepatic triglycerides (Fig. 3f).

Taken together, these findings indicate that miR-26a contributes to ethanol-induced autophagy, thereby playing a role in alleviating ethanol-induced hepatic steatosis and liver injury.

### miR-26a targets several genes involved in autophagy

**Fig. 4:**
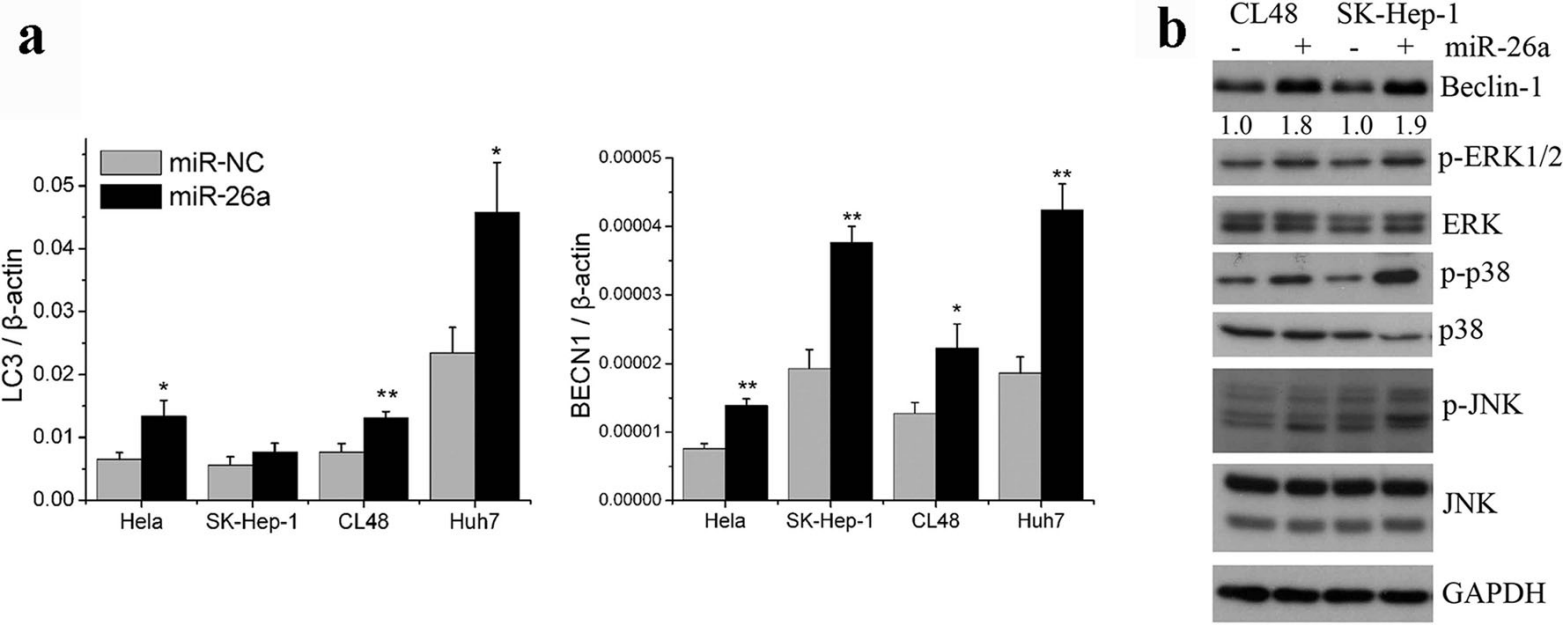
miR-26a increases the expression of Beclin-1 by the activation of MAPKs. **a** Expression levels of Beclin-1 and LC3 were measured by qRT-PCR in cells transfected with miR-26a for 24 h. **b** Cells were transfected with miR-26a or scramble miRNA (miR-NC) for 48 h. Lysates of treated cells were subjected to immunoblotting. Beclin-1 was quantified by ImageJ densitometric analysis and normalized using GAPDH. Results shown are the mean ± SD of at least three independent experiments. *P < 0.05; **P < 0.01 compared with cells transfected with scramble miRNA

To understand the mechanism by which miR-26a enhances autophagy, we evaluated the effect of miR-26a on the expression of the main autophagy-related genes, including Beclin-1 and LC3. As shown in Fig. 4a, the mRNA levels of Beclin-1 and LC3 were significantly increased in cells transfected with miR-26a. Beclin-1 plays a central role in autophagy and moderately elevated Beclin-1 results in cytoprotective autophagy [29]. Beclin-1 is regulated by many factors, including MAPKs [30]. We therefore measured MAPK signaling in miR-26a transfected cells. The results demonstrated that miR-26a enhanced the phosphorylation of ERK1/2, p38, and JNK (Fig. 4b). To better understand the underlying mechanisms by which miR-26a activates MAPKs, potential miR-26a target genes were searched with TargetScan 6.2. We found that miR-26a can potentially target DUSP4 and DUSP5, two negative regulators of the phosphorylation of ERK1/2, JNK, and p38 [31, 32].

**Fig. 5:**
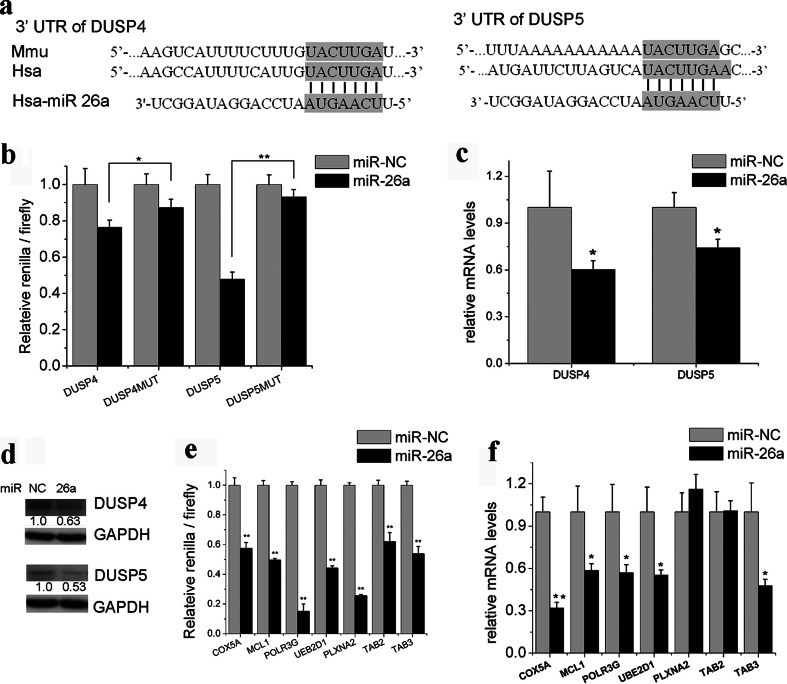
miR-26a targets several genes involved in the induction of autophagy. **a** Diagram of the 3′-UTRs of DUSP4 and DUSP5 in different species. **b** Luciferase reporter assay of psicheck2.2 with 3′-UTR fragments of DUSP4 and DUSP5. **c**, **d** mRNA and protein levels of DUSP4 and DUSP5 in Huh7 cells transfected with miR-26a. **e** Luciferase reporter assay of psicheck2.2 with 3′-UTR fragments of potential targets. **f** Real-time PCR analysis of miR-26a target genes after transfection with the miR-26a precursors. Results shown are the mean ± SD. *P < 0.05; **P < 0.01 compared with cells transfected with scramble miRNA

To validate the predicted binding sites of miR-26a in the 3′-UTR of DUSP4 and DUSP5 (Fig. 5a), we examined miR-26a’s interaction with this domain by luciferase reporter assay in Hela cells using a psicheck2.2 vector containing the 3′-UTR of the target genes or a control psicheck2.2 vector containing the same 3′-UTR with mutated miR-26a seed nucleotides. miR-26a precursors repressed the luciferase activities of the vector containing the wild-type 3′-UTR of DUSP4 by more than 23 % and the wild-type 3′-UTR of DUSP5 by more than 52 %. By contrast, mutation of the seed sequence abolished this repression (Fig. 5b). Furthermore, we also found that the transfection of miR-26a precursors resulted in a significant decrease in both the mRNA and protein levels of these two target genes in Huh7 cells (Fig. 5c, d). To further confirm the functional roles of DUSP4 and DUSP5 in autophagy, siRNAs against DUSP4 and DUSP5 were used to knock down these genes in SK-Hep-1 cells. As shown in supplementary Fig. 5, knockdown of DUSP4 or DUSP5 increased the phosphorylation of ERK1/2, p38, and JNK and resulted in the accumulation of LC3-II or LC3 puncta in SK-Hep-1/tf-LC3 cells (supplementary Figs. 5 and 6). These results strongly suggest the functional importance of DUSP4 and DUSP5 as miR-26a targets.

miRNAs usually repress the expression of multiple genes involved in the same pathway. Therefore, we evaluated other predicted miR-26a target genes that are potentially involved in autophagy regulation, including MCL1 [33], TAB2, and TAB3 [34]. Recently, Lipinski et al. identified a number of genes that negatively regulate autophagy using a siRNA screen [35]. Among these negative regulators of autophagy, we identified several potential targets of miR-26a. A luciferase reporter assay demonstrated that COX5A, MCL1, POLR3G, UBE2D1, PLXNA2, TAB2, and TAB3 can be targeted by miR-26a directly (Fig. 5e). As a result, transfection of miR-26a precursors led to a significant decrease in the endogenous mRNA levels of COX5A, MCL1, POLR3G, UBE2D1, and TAB3 in Huh7 cells (Fig. 5f).

## Discussion

miRNAs such as miR-30a, 101, 375, 204, 130a, and 376b have recently been determined to modulate autophagy by targeting some members of ATGs and their regulators at different autophagic stages. Eventually, all these miRNAs inhibit the autophagic process [10, 13, 36–38]. Meanwhile, some reports have shown that miRNAs could promote autophagy by targeting negative regulators of autophagy. For example, microRNA-155 promotes autophagy to eliminate intracellular mycobacteria by targeting Rheb [14], and hypoxia-induced miR155 is a potent autophagy inducer by targeting multiple players in the MTOR pathway [39]. microRNA-18a upregulates autophagy and ataxia telangiectasia mutated gene expression in HCT116 colon cancer cells [40]. microRNA-100 promotes the autophagy of hepatocellular carcinoma cells by inhibiting the expression of mTOR and IGF-1R [41]. In the present study, we show that miR-26a promotes autophagy and that overexpression of miR-26a in mouse liver alleviates ethanol-induced hepatic steatosis and liver injury. To the best of our knowledge, this is the first study to demonstrate that miR-26a can promote cytoprotective autophagy.

Most of the cells in the human body have a basal level of autophagy. Autophagy is an important physiological mechanism that may serve as a temporary survival mechanism during periods of metabolic stress. Beclin-1 has been well characterized as playing a pivotal role in autophagy, and its dysfunction has been implicated in many disorders, including embryonic development, cancer, and neurodegeneration. Previous studies revealed that mammalian species fail to survive in the absence of Beclin-1 and develop cancers with the low expression of Beclin-1. Moderately elevated Beclin-1 expression induces cytoprotective autophagy, while overexpression activates destructive autophagy and cell death [29]. In our studies, Beclin-1 expression in miR-26a transfected cells is moderately increased (less than threefold in Fig. 4); thus, we expect that miR-26a-mediated autophagy is cytoprotective. This hypothesis was confirmed by the in vivo ethanol binge model. Overexpression of miR-26a in the liver alleviates ethanol-induced hepatic steatosis and liver injury, and this cytoprotective effect was abolished by the autophagy inhibitor CQ (Fig. 2). To date, a few studies have reported the use of rapamycin to induce autophagy as a potential therapeutic approach [4, 42]. However, rapamycin has strong immunosuppression activity. Our study proposes a potential usage for miR-26a as an autophagy inducer for the treatment of autophagy-related diseases, such as ethanol-induced hepatic steatosis and liver injury.

The sober-up time (i.e., time to restore the righting reflex) of L-TG and WT mice after ethanol binge was approximately 2 and 3.5 h, respectively. This difference in Fig. 2a is fascinating and statistically significant. The sober-up time likely depends on the rate of ethanol metabolism. It is well known that ethanol is almost completely absorbed from the gastrointestinal tract and eliminated mainly in hepatocytes [43]. The ethanol binge leads to the accumulation of lipid droplets in hepatocytes that results in the development of liver steatosis and subsequent hepatocyte dysfunction, which impairs the metabolism of alcohol. Ding et al. reported that autophagy constitutes an effective defense mechanism against ethanol-induced hepatotoxicity by removing damaged mitochondria and accumulated fatty acids [4]. Our results also show that miR-26a could significantly reduce hepatic steatosis via the autophagic degradation of LDs, thereby indirectly promoting ethanol metabolism and shortening the ethanol binge-induced sober-up time. Moreover, this reduction in sober-up time by miR-26a could be autophagy-dependent because CQ, a widely used autophagy inhibitor, abolished the protective effect of miR-26a (Fig. 2a).

We observed that miR-26a transfection led to the activation of ERK1/2, p38, and JNK (Fig. 4b), which are the best-studied MAP kinases in eukaryotic cells. MAPKs are a widely conserved family of serine/threonine protein kinases implicated in many cellular programs, such as cell proliferation, differentiation, and apoptosis. An increasing number of studies have suggested that MAPKs also play important roles in modulating autophagy. One crucial mechanism by which MAPKs contribute to autophagy is the increase of Beclin-1 expression [29, 44, 45]. The present study demonstrated that miR-26a suppressed DUSP4 and DUSP5, two negative regulators of MAPKs [31], by directly interacting with their 3′-UTRs, which resulted in activated MAPKs and moderately enhanced the expression of Beclin-1 (Fig. 4b). As mentioned above, a moderate increase in Beclin-1 expression results in cytoprotective autophagy. Therefore, it is likely that miR-26a regulates a network of targets that influence autophagy through mechanisms and pathways converging at the level of Beclin-1. Transcription factor EB (TFEB) is an important transcription factor that promotes autophagy by regulating coordinated lysosomal expression and regulation gene expression. Previous studies suggest that TFEB transcriptional activity can be downregulated by phosphorylation via ERK1/2 [46]. However, when we checked the TFEB changes in cells transfected with miR-26a (supplementary Fig. 7), miR-26a overexpression did not induce TFEB nuclear translocation, which indicates that TFEB may not be essential in miR-26a-mediated autophagy. We also identified several other autophagy negative regulators as miR-26a targets, including COX5A, POLR3G, UBE2D1, and PLXNA2 (Fig. 5e). Further investigation is required to determine how these target genes are involved in miR-26a’s regulation of autophagy.

**Fig. 6:**
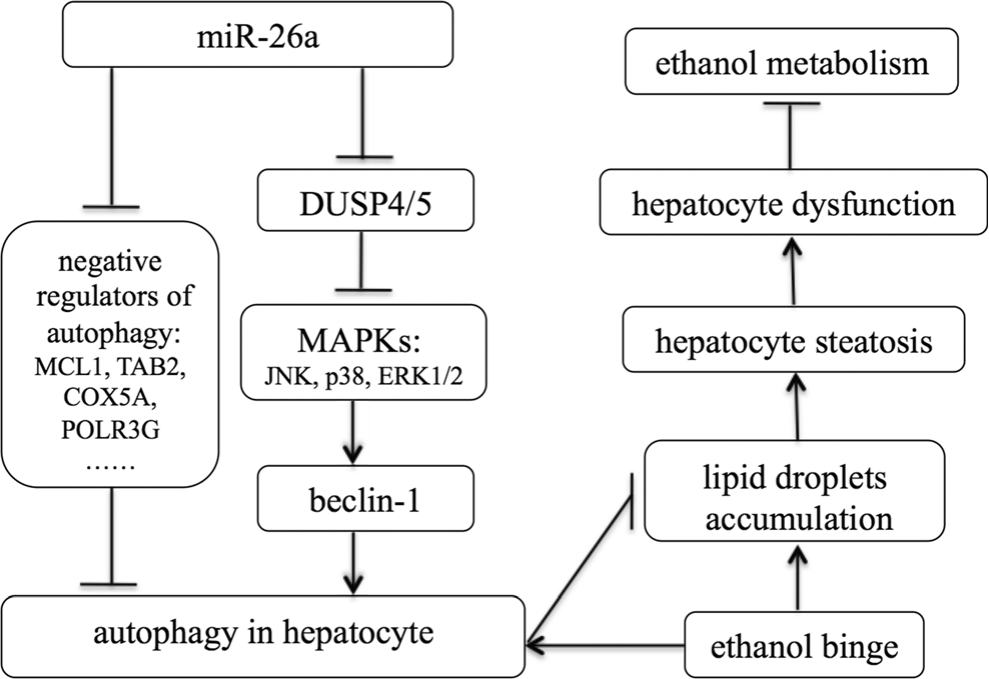
Summary diagram: hepatic overexpression of miR-26a in mice alleviates ethanol-induced hepatic steatosis and liver injury by augmenting autophagic degradation of lipid droplets. Overexpression of miR-26a increases the expression of the autophagy mediator Beclin-1, which is regulated by mitogen-activated protein kinases. DUSP4 and DUSP5, two MAPK inhibitors, were identified as direct targets of miR-26a. Several other negative regulators of autophagy, such as MCL1, TAB2, COX5A, and POLR3G, were also identified as potential targets of miR-26a. Forced expression of miR-26a in the liver can alleviate ethanol-induced hepatic steatosis and liver injury by augmenting the autophagic degradation of lipid droplets in hepatocytes

In summary, our findings demonstrated that miR-26a could enhance autophagy through the inhibition of some negative regulators of autophagy. Forced expression of miR-26a in the liver can alleviate ethanol-induced hepatic steatosis and liver injury by augmenting the autophagic degradation of lipid droplets in hepatocytes (Fig. 6). Therefore, enhancement of autophagy by miR-26a may provide a potential therapeutic strategy to protect the liver from ethanol and other agent-induced liver injuries.

## Electronic supplementary material

ESM 1 (PDF 419 kb)

## Abbreviations

3′-UTR: 3′ untranslated region
miRNAs: MicroRNAs
miR-26a: MicroRNA 26a
MAPKs: Mitogen-activated protein kinases
LDs: Lipid droplets
mRNAs: Messenger RNAs
CQ: Chloroquine
tf-LC3: Tandem fluorescent-tagged LC3
PCR: Real-time polymerase chain reaction
ALT: Alanine aminotransferase
TEM: Transmission electron microscopy
