# Supplementary material for: miR-26a enhances autophagy to protect against ethanol-induced acute liver injury
Source: J Mol Med (Berl). 2015 Apr 17;93(9):1045–55. doi: 10.1007/s00109-015-1282-2 (PMC4577542; doi:10.1007/s00109-015-1282-2)
Supplement: Supplementary file 1 — (PDF 419 kb) [file 109_2015_1282_MOESM1_ESM.pdf]

J Mol Med 2015

miR-26a Enhances Autophagy to Protect Against Ethanol-Induced Acute Liver Injury

Weidong Han[1,2*], Xianghui Fu[2,3*], Jiansheng Xie[1], Zhipeng Meng[2], Ying Gu[2], Xichun Wang[2], Ling Li[2], Hongming Pan[1], and Wendong Huang[2]

[1]Department of Medical Oncology, Sir Run Run Shaw Hospital, College of Medicine, Zhejiang University, Hangzhou, Zhejiang, China; [2]Division of Molecular Diabetes Research, Department of Diabetes and Metabolic Diseases Research, Beckman Research Institute, City of Hope National Medical Center, Duarte, CA; [3]Division of Endocrinology and Metabolism, State Key Laboratory of Biotherapy/Collaborative Innovation Center of Biotherapy, West China Hospital, Sichuan University, Chengdu, Sichuan, China.

Contact Information

Corresponding authors: Hongming Pan, 3 East Qingchun Road, Hangzhou 310016, China. Tel.: +86 571 86006922; Fax: +86 571 86436673; E-mail: hongmingpan@gmail.com; or Wendong Huang, City of Hope National Medical Center, 1500 E. Duarte Road, Duarte, CA 91010. Tel.: 626-256-4673; Fax: 626-256-8704; E-mail: whuang@coh.org; or Xianghui Fu, Email: xfu@scu.edu.cn.

Supplementary Materials and Methods

Cell Culture and Transfection Hela, HepG2, SK-Hep-1, CL48 cells were purchased from the American Type Culture Collection. Huh7 cells were kindly provided by Dr. Clifford J. Steer. Hela, HepG2 and SK-Hep-1 cells were cultured in modified Eagle's medium supplemented with 10% fetal bovine serum. Huh7 cells were cultured in RPMI 1640 medium supplemented with 10%

fetal bovine serum. Normal human embryonic liver cell line CL-48 cells were cultured in high glucose DMEM supplemented with 10% fetal bovine serum. The mRFP-GFP tandem fluorescent-tagged LC3 (tf-LC3) plasmid was obtained from Addgene. Renilla luciferase (RLuc)–LC3^{WT} and RLuc–LC3^{G120A} were kindly provided by Dr. Marja Jäättelä. Transfection was performed using Attractene (Qiagen, Valencia, CA) according to the manufacturer's instructions. Stable transformants were selected in a complete medium containing 500 μg/ml G418 (Sigma, St. Louis, MO).

Real-Time Polymerase Chain Reaction Analysis For the analysis of miR-26a expression, reverse transcription was performed with Superscript III reverse transcriptase (Invitrogen, Carlsbad, CA). Real-time polymerase chain reaction (PCR) was performed using the Power SYBR Green PCR Master Mix protocol (Applied Biosystems, Foster City, CA). 5S RNA was used to normalize the expression levels of miRNAs. Sequences of the primers for reverse transcription and PCR are provided in the Supporting Information. To analyze mRNAs, reverse transcription was performed with Superscript III reverse transcriptase and Oligo(dT)20 at 50 °C for 1 hour. Primers for the miR-26a target genes were provided in the Supporting Information Table 1. Gene expression levels for mRNAs were standardized using β-actin (Ambion, Austin, TX).

Immunoblot Analysis Proteins were separated by 10% sodium dodecyl sulfate– polyacrylamide gel electrophoresis and transferred to nitrocellulose membranes. After blocking in 5% nonfat milk, membranes were incubated with the following primary antibodies: anti–LC3B, anti-p62, anti-ERK1/2, anti-phospho-ERK1/2, anti-JNK, anti-phospho-JNK, anti-p38, anti-phospho-p38, anti-Beclin-1, anti-GAPDH and anti-ATG7 from Cell Signaling Technology, Inc. (Danvers, MA); and anti-DUSP4 and anti-DUSP5 from Santa Cruz Biotechnology (Santa Cruz, CA). Membranes were washed and exposed to peroxidase-conjugated secondary antibodies (Amersham Bioscience,

UK).

Confocal Microscopy Stable transformants were plated on noncoated cover slips and then cultured for 24 hours. These cells were transfected with miR-26a or scramble miRNA (miR-NC) for 48 hours and then fixed with 4% paraformaldehyde in PBS at room temperature. After fixation, cells were mounted with DAPI and viewed under a Zeiss LSM 510 confocal microscope system (Carl Zeiss, Jena, Germany).

Transmission Electron Microscopy Cells or liver tissues were washed and fixed for 30 min with 2.5% glutaraldehyde. The samples were then treated with 1.5% osmium tetroxide, dehydrated with acetone and embedded in Durcupan resin. Thin sections were poststained with lead citrate and examined using the FEI Tecnai transmission electron microscope.

Oil Red Stain For the detection of neutral lipids, mice liver cryosections were stained using the Oil Red O technique. Briefly, liver cryosections were fixed for 10 min in 60% isopropanol, stained with 0.3% Oil Red O in 60% isopropanol for 30 min, and subsequently washed with 60% isopropanol. Sections were counterstained with Gill's hematoxylin, washed with acetic acid solution (4%), and mounted with aqueous solution.

Immunofluorescent Confocal Laser Microscopy Cells were collected, fixed and permeabilized with 1% CHAPS buffer (150 mM NaCl, 10 mM HEPES, 1.0% CHAPS) at room temperature for 10 min. They were then incubated with anti-TFEB (ab122910, Abcam, UK) for 2 h at room temperature, washed with PBS, and incubated for another 45 min with FITC-conjugated goat anti-goat IgG (Beyotime, China). Cell nuclei were then stained with DAPI (Sigma). Samples were examined under a Zeiss LSM 710 confocal microscope system (Carl Zeiss, Germany). The image was processed with ZEN LE software.

Supporting Information

Primer for miR-26a reverse transcription:

5’-CATGATCAGCTGGGCCAAGAAGCCTATCCTGG-3’

Real-time PCR primers for miR-26:

F: 5’-TT+CA+AGTAATCCAGGA-3’

R: 5’-CATGATCAGCTGGGCCAAGA-3’

Note: "+" means LNA (locked nucleic acid) substitution

Table 1. Information of Real-time PCR primers

Genes	Real time PCR primers sequence
DUSP4	F: 5’-GGCGGCTATGAGAGGTTTTCC-3’; R: 5’-TGGTCGTGTAGTGGGGTCC-3’
DUSP5	F: 5'-TGTCGTCCTCACCTCGCTA-3'; R: 5'-GGGCTCTCTCACTCTCAATCTTC-3'
COX5A	F: 5'-GCCAGATATAGATGCCTGGGA-3';
	R: 5'-ACAACCTCTAGGATACGAACTGT-3'
MCL1	F: 5’-GCCAAGGACACAAAGCCAAT-3’; R: 5’-AACTCCACAAACCCATCCCA-3’
POLR3G	F: 5'-GAGGACGTGCTGCTTATACCT-3'; R: 5'-CTGTTCTGCGGCATCATCGT-3'
UBE2D1	F: 5'-TAGCGCATATCAAGGTGGAGT-3'; R: 5'-TGGTGACCATTGTGACCTCAG-3'
PLXNA2	F: 5'-CTGAGAATCGTGACTGGACCT-3'; R: 5'-GCTTATAGACCCGGTTGATGG-3'
TAB2	F: 5’-GCATTCTGGCTGGGTAT-3’; R: 5’-GCTGATTTGGCTGTTGA-3’
TAB3	F: 5’-TGTACTCCATCACCATCTCCT-3’; R: 5-TGCTTTGCTAACCTCTCCAT-3’

Supplementary Figure and Figure Legends

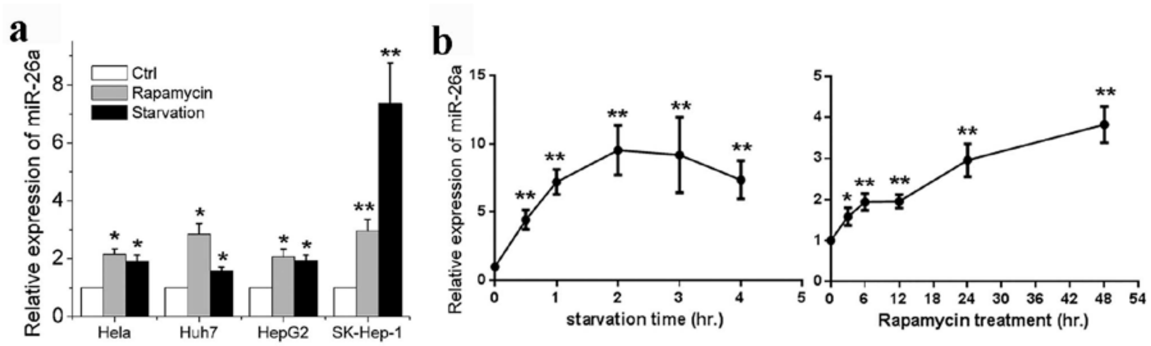

Supplementary Fig. 1 Effects of HBSS and rapamycin on endogenous miR-26a expression

a. Hela, Huh7, HepG2 and SK-Hep-1 cells were treated with HBSS for 4 hours or 125 nM rapamycin for 24 hours. **b.** SK-Hep-1 cells were treated with HBSS or 125 nM rapamycin for different time intervals. At the end of the treatments, endogenous miR-26a expression was analyzed by qRT-PCR. 5S RNA was used as the internal control. Results shown are the mean ± SD of at least three independent experiments. *P < 0.05, ** P < 0.01 vs. control.

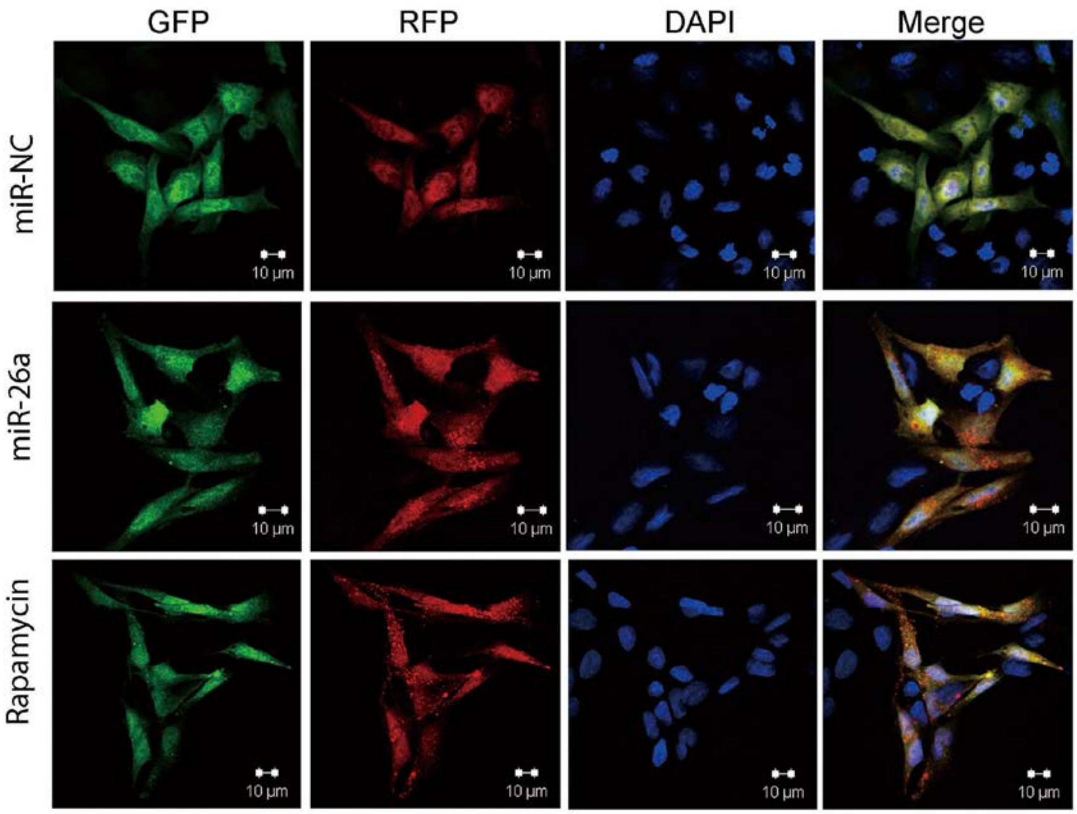

Supplementary Fig. 2 miR-26a overexpression promotes autophagic flux

Representative confocal images of SK-Hep-1/tf-LC3 cells transfected with miRNAs or treated with rapamycin were indicated. Scale bar = 10 μm.

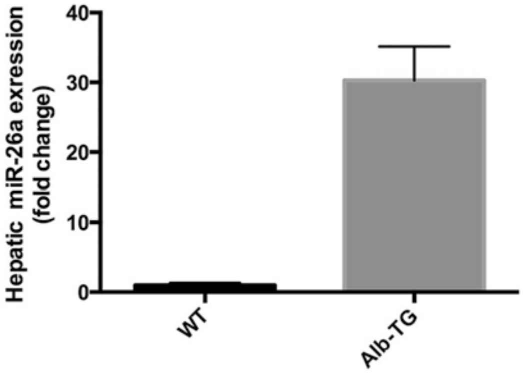

Supplementary Fig. 3 Generation of liver-specific transgenic mice (L-TG)

The expression of miR-26a in mice liver was analyzed by qRT-PCR. 5S RNA was used as the internal control.

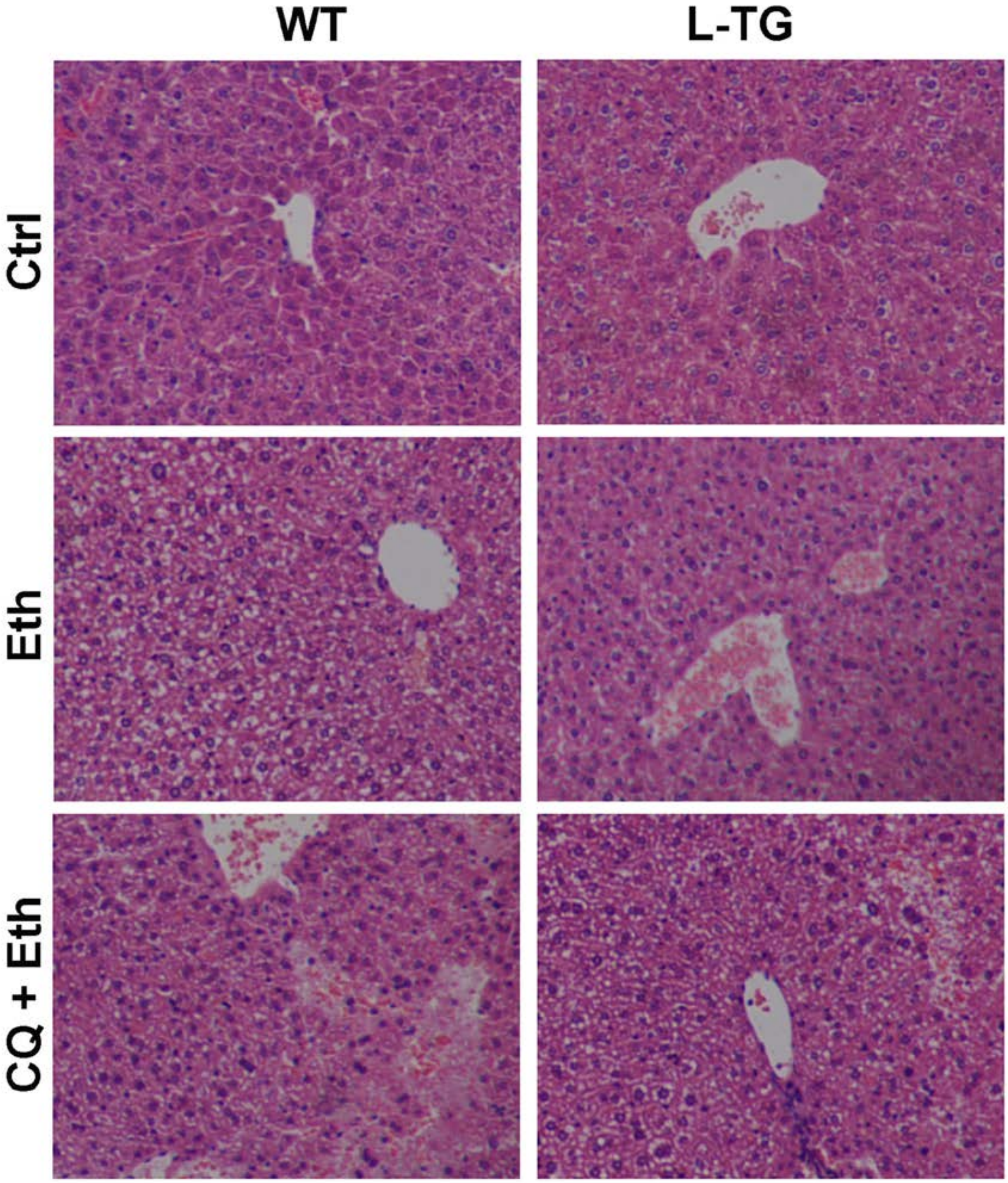

Supplementary Fig. 4 Forced expression of miR-26a alleviates ethanol-induced hepatic

steatosis and liver injury

Representative H&E staining images of mice liver after ethanol binge in the presence or absence of autophagy inhibitor CQ were indicated.

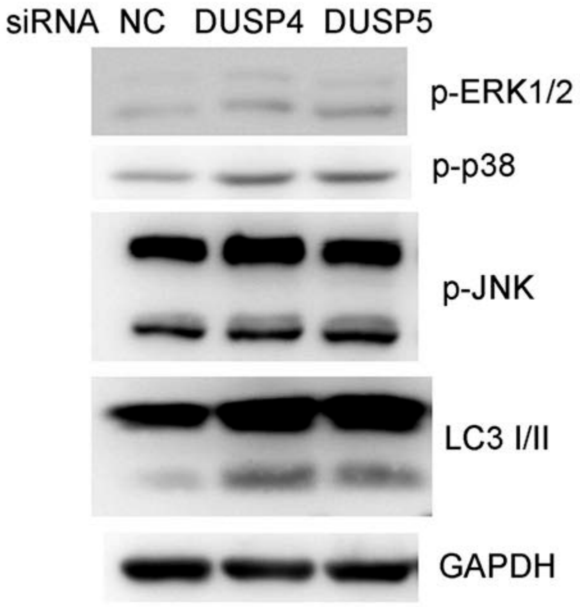

Supplementary Fig. 5 Knockdown of DUSP4 or DUSP5 leads to the activation of MAPKs and the upregulation of LC3II

SK-Hep-1 cells were transfected with siRNA against DUSP4 or DUSP5 for 48 hours. Lysates of the treated cells were subjected to Western blotting.

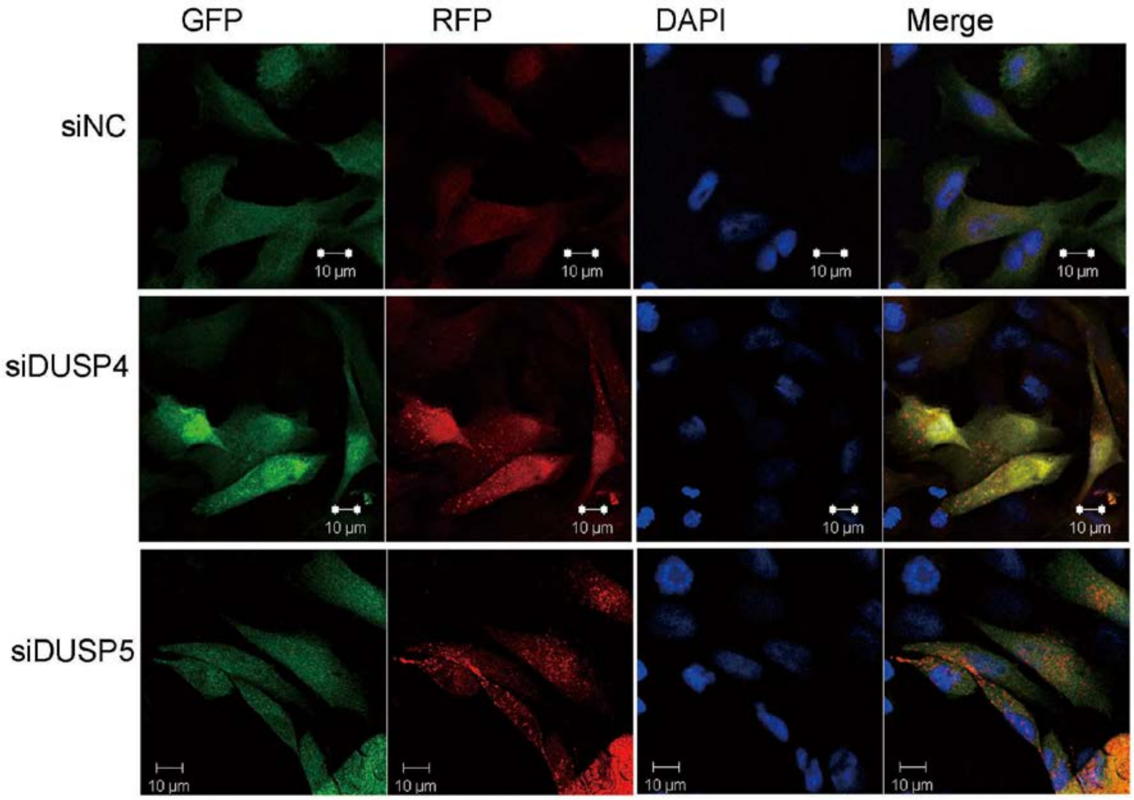

Supplementary Fig. 6 Knockdown of DUSP4 or DUSP5 promotes autophagy

Representative confocal images of SK-Hep-1/tf-LC3 cells transfected with siRNA against DUSP4 or DUSP5 were indicated. Scale bar = 10 μm.

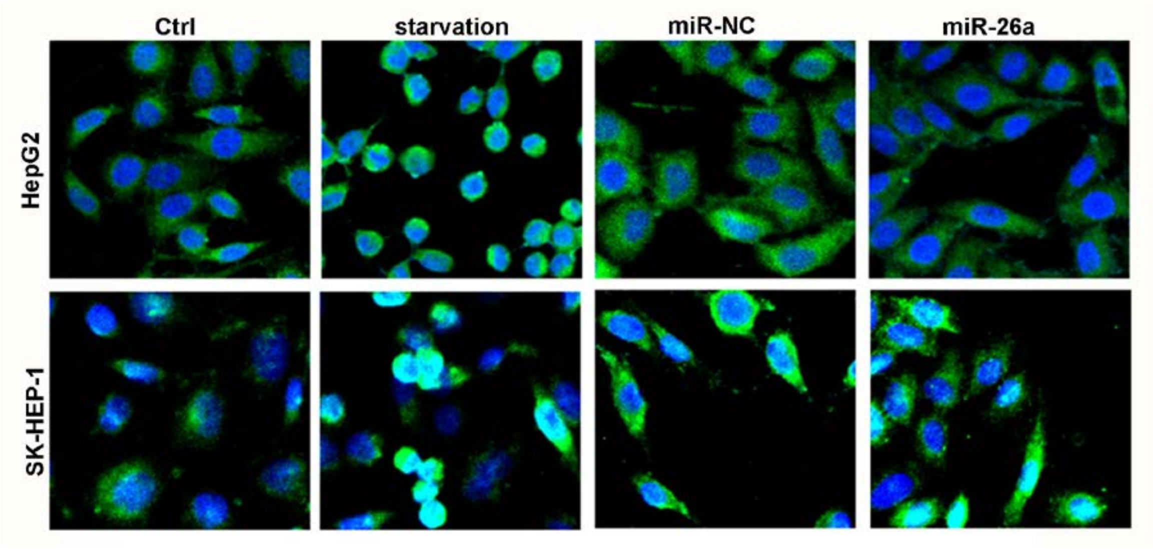

Supplementary Fig. 7 TFEB is not involved in miR-26a-mediated autophagy

Representative confocal images of SK-Hep-1 and HepG2 cells transfected with miRNAs or treated with starvation were indicated. Starvation for 2 hours was use a positive control for autophagy and TFEB nuclear translocation.
